# Effect of high-intensity interval training on peak oxygen uptake, quality of life, and ventricular arrhythmias in patients with an implantable cardioverter defibrillator: a randomized controlled trial

**DOI:** 10.1093/ehjopen/oeag058

**Published:** 2026-04-13

**Authors:** Mathias Nyman, Ole Christian Mjølstad, Ane Cecilie Dale, Brage Høyem Amundsen, Ole Rossvoll, Ulrik Wisløff, Jan Pål Loennechen

**Affiliations:** Clinic of Cardiology, St.Olavs Hospital, Trondheim University Hospital, Postboks 3250 Torgarden, 7006 Trondheim, Norway; Department of Circulation and Medical Imaging, Norwegian University of Science and Technology (NTNU), Postboks 8905, 7491 Trondheim, Norway; Clinic of Cardiology, St.Olavs Hospital, Trondheim University Hospital, Postboks 3250 Torgarden, 7006 Trondheim, Norway; Department of Circulation and Medical Imaging, Norwegian University of Science and Technology (NTNU), Postboks 8905, 7491 Trondheim, Norway; Clinic of Cardiology, St.Olavs Hospital, Trondheim University Hospital, Postboks 3250 Torgarden, 7006 Trondheim, Norway; Clinic of Cardiology, St.Olavs Hospital, Trondheim University Hospital, Postboks 3250 Torgarden, 7006 Trondheim, Norway; Department of Circulation and Medical Imaging, Norwegian University of Science and Technology (NTNU), Postboks 8905, 7491 Trondheim, Norway; Clinic of Cardiology, St.Olavs Hospital, Trondheim University Hospital, Postboks 3250 Torgarden, 7006 Trondheim, Norway; Department of Circulation and Medical Imaging, Norwegian University of Science and Technology (NTNU), Postboks 8905, 7491 Trondheim, Norway; Department of Circulation and Medical Imaging, Norwegian University of Science and Technology (NTNU), Postboks 8905, 7491 Trondheim, Norway; Clinic of Cardiology, St.Olavs Hospital, Trondheim University Hospital, Postboks 3250 Torgarden, 7006 Trondheim, Norway; Department of Circulation and Medical Imaging, Norwegian University of Science and Technology (NTNU), Postboks 8905, 7491 Trondheim, Norway

**Keywords:** High-intensity interval training, Ventricular arrhythmia, ICD, VO_2_peak, Quality of life

## Abstract

**Aims:**

Exercise is effective in preventing and treating cardiovascular disease. High-intensity interval training (HIIT) has shown promising effects on cardiorespiratory fitness and quality of life (QoL). However, evidence of risks and beneficial effects of HIIT in patients at high risk of ventricular arrhythmias (VA) is limited. This study evaluated the effects of HIIT on peak oxygen uptake (VO_2_peak), QoL, and the burden of VA in patients with an implantable cardioverter defibrillator (ICD).

**Methods and results:**

Fifty-six ICD patients with coronary artery disease (CAD) or non-ischaemic dilated cardiomyopathy (DCM) were randomized to a 12-week supervised HIIT programme with intervals at 85–95% of maximum heart rate, or to usual activity (control). Primary outcomes were changes in VO_2_peak and QoL. Secondary outcomes included changes in VA burden, with or without ICD therapy. High-intensity interval training increased VO_2_peak by 7.0% vs. no change in the control group, with a between-group difference of 1.7 mL/kg/min (95% confidence interval, 0.7–2.6; *P* < 0.001). After correction for multiple testing, HIIT improved QoL on the SF-36 health change domain, while most other domains showed favourable but non-significant trends. Clinically relevant VA occurred in two patients during baseline exercise testing and in two patients during HIIT. Sustained ventricular tachycardia incidence was lower in the HIIT group (*P* = 0.037), although the number of events was small and unevenly distributed.

**Conclusion:**

In ICD patients with CAD or non-ischaemic DCM, a supervised 12-week HIIT programme significantly improved exercise capacity and QoL. However, its overall impact on VA remains inconclusive, and the risk of exercise-induced arrhythmias remains a concern.

## Graphical Abstract

**Graphical Abstract:**
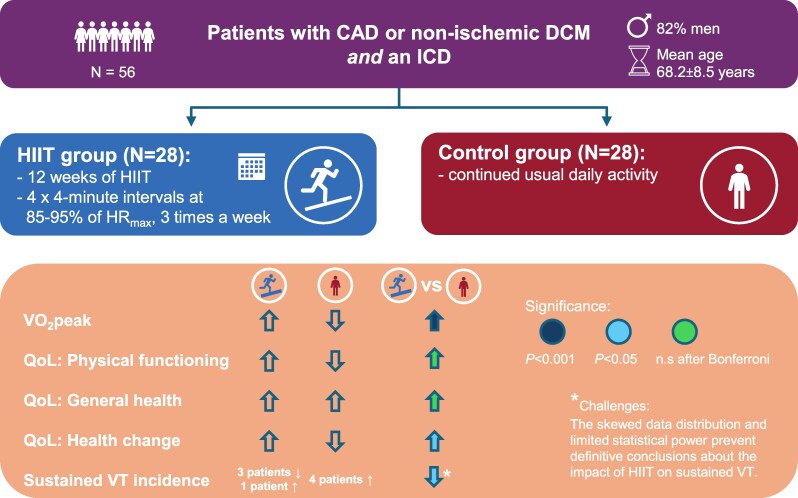

## Introduction

Extensive research highlights exercise as an effective preventive and therapeutic approach for most cardiovascular diseases and their associated risk factors. Particularly in patients with coronary artery disease (CAD) and heart failure (HF), exercise reduces cardiovascular mortality, decreases hospitalizations, and improves cardiorespiratory fitness and quality of life (QoL).^1–3^

For decades, high-intensity interval training (HIIT) has gained growing attention as an alternative exercise regimen, showing superior effects in improving cardiorespiratory fitness compared with the conventional approach of moderate-intensity continuous training.^4–6^ However, similar improvements have not been observed in terms of QoL.^7,8^

The safety profile of HIIT for patients at high risk of major adverse cardiovascular events appears promising.^9,10^ Nevertheless, comprehensive documentation of the benefits and possible risks of HIIT in patients with high risk of ventricular arrhythmia (VA), particularly those with an implantable cardioverter defibrillator (ICD) for primary or secondary prevention, remains limited.^11^

While current guidelines on exercise emphasize the importance of adhering to recommendations related to the underlying disease, there is a limited level of recommendation and evidence on the participation in high-intensive exercise for individuals with ICDs.^11^ More research is needed to understand how HIIT impacts the risks of both VA and inappropriate ICD shocks.

A meta-analysis of six trials showed improvement in cardiorespiratory fitness and a reduced likelihood of ICD shocks among patients with HF and ICD following exercise training, but study populations, study design, and exercise intensity varied across these trials.^12^ In a non-randomized study, an HIIT programme targeting 85% of maximum heart rate (HR_max_) during intervals proved feasible and effective in HF patients with an ICD or a cardiac resynchronization therapy-defibrillator (CRT-D).^13^ Beyond structured exercise programmes, international guidelines based on prospective observational data suggest that athletes with ICDs may participate in vigorous and competitive sports after individualized evaluation.^14,15^ This aligns with findings that some ICD patients are highly motivated to lead an active lifestyle, including strenuous exercise.^16^ However, patients with ICDs often adjust their daily routines and avoid certain activities due to fear of ICD shocks or uncertainty about their physical endurance, potentially impacting their QoL.^17,18^

Given these considerations, the primary objectives of this study were to evaluate the effects of HIIT on exercise capacity and QoL in patients with an ICD due to CAD or non-ischaemic dilated cardiomyopathy (DCM). Secondary outcomes included changes in the burden of premature ventricular contractions (PVCs), non-sustained ventricular tachycardia (NSVT), and sustained ventricular tachycardia (VT). We hypothesized that HIIT would improve exercise capacity and QoL in these patients.

## Methods

### Study sample

All patients, a total of 859 individuals (81% men), who underwent an ICD implantation at the Clinic of Cardiology, St. Olavs hospital, Trondheim, Norway, between January 2014 and September 2021, were assessed for eligibility in this study. Enrolment occurred between September 2019 and January 2022. The inclusion criteria were age ≥18 years with a diagnosis of CAD or non-ischaemic DCM as the indication for ICD or CRT-D implantation, for either primary or secondary prevention indication. Exclusion criteria were comorbidity that prevented participation in regular HIIT, severe valvular heart disease, symptomatic CAD, or unstable symptomatic arrhythmia. Other exclusion criteria were planned surgery within 6 months, job-related or logistical constraints preventing participation in the training programme, including residing more than 1 h's drive from the training location, and involvement in training with intensity levels equivalent to HIIT twice per week or more. Patients who experienced sustained VT, symptomatic NSVT, or showed evidence of severe cardiac ischaemia during the baseline treadmill cardiopulmonary exercise test (CPET) were also excluded. Because recruitment was slightly lower than anticipated, enrolment concluded with 56 patients, short of the planned sample size of 60.

### Study design

The study was a single-centre, randomized controlled trial. Following baseline data collection, patients were stratified based on the underlying cause of device implantation (CAD or DCM) and randomized in a 1:1 ratio to participation in a supervised HIIT programme or to a control group continuing usual exercise activity. Stratification and randomization procedures were conducted using WebCRF, a web-based tool developed and administered by the Unit of Applied Clinical Research, Faculty of Medicine and Health Sciences, Norwegian University of Science and Technology (NTNU), Trondheim, Norway. This tool employed permuted block randomization, with specific details undisclosed to the researchers.

The primary investigator was in principle blinded to the intervention during data collection and analyses, though full blinding was challenging due to the small sample size.

The study was conducted in accordance with the principles set forth in the Helsinki Declaration, approved by the regional ethics committee (no. 2018/1592/REK sør-øst B), and registered on Clinicaltrials.gov (identifier: NCT04070300).

An internal, independent clinical safety advisor was consulted on adverse events. Also, an external clinical safety committee was established to evaluate any serious adverse events.

All participants provided written and oral informed consent.

Study data are available upon reasonable request from the corresponding author.

### Exercise testing

An individualized CPET was performed to measure the peak oxygen uptake (VO_2_peak) and HR_max_ for training intensity purposes, as well as to screen for potential exercise-induced VA or symptomatic myocardial ischaemia. Familiarization to treadmill walking/running was done during 10–15 min of warm-up. Patients were instructed to exert themselves to their maximum capacity, as previously described.^19^ The follow-up test was conducted under the supervision of an exercise physiologist who was blinded to the intervention. To avoid inappropriate ICD shocks, tachycardia therapy was temporarily deactivated. The programmer device and a manual external defibrillator were readily available, with an experienced cardiologist present during testing.

Testing was performed on a treadmill (PPS 55med; Woodway, Waukesha, USA) using a protocol with individualized band speed and inclination. Heart rate (HR), a 12-lead ECG (Cardio 100 BT; Custo Med, Ottobrunn, Germany), and ventilation/gas exchange data (Metalyzer II; Cortex Biophysik, Leipzig, Germany) were continuously recorded. The test was considered complete when we observed VO_2_ levelling off despite increased workload. Additional criteria included a respiratory exchange ratio (RER) ≥ 1.05 and/or a Borg’s rate of perceived exertion (RPE) ≥ 17.^20^ VO_2_peak was defined as the mean of the three highest 10 s measurements obtained, and HR_max_ as peak measured HR +5 b.p.m.^21^ Tachycardia detection was reactivated after testing.

### High-intensity interval training protocol

The 12-week HIIT programme took place at the NextMove Core facility of the Cardiac Exercise Research Group (CERG), situated at St Olavs Hospital, Trondheim, Norway. The training programme consisted of uphill walking/running on a treadmill (TL200; GymSport, Trondheim, Norway). Training sessions were performed three times weekly in groups of two to four participants under the supervision of an exercise physiologist. Safety was ensured by the hospital’s cardiac emergency response plan. The structured HIIT protocol began with a 10 min warm-up at 60–70% of HR_max_, corresponding to an RPE of 11–13 on Borg’s scale. This was followed by four 4 min intervals, targeting 85–95% of HR_max_ and/or an RPE of 15–17, each separated by a 3 min active recovery period at 60–70% of HR_max_ and/or an RPE of 11–13. Each session ended with a 5 min cool-down, totalling 40 min of exercise.

Participants wore HR monitors (Polar H10; Polar Electro, Kempele, Finland) to ensure adherence to the prescribed exercise intensity. The instructor monitored the participants, ensuring their compliance with desired intensity levels and encouraging speed or inclination increases to match their improved exercise capacity, keeping the relative exercise intensity constant. As a safety precaution, participants remained at the facility for 5–10 min of post-exercise observation.

### Control group instructions

Patients in the control group did not receive specific instructions on a minimum level of physical activity, other than to maintain their usual activity level if desired. However, they were instructed not to initiate any new exercise programmes, including HIIT.

### Quality of life

Health-related QoL was assessed using the Norwegian version of the RAND 36-item Short-Form (SF-36) patient-reported questionnaire.^22^ SF-36 measures various aspects of QoL and includes the following subscales: physical functioning, role limitations due to physical health, role limitations due to emotional problems, energy/fatigue, emotional well-being, social functioning, pain, general health, and health change. Scores for each domain range from 0 to 100, with a higher score indicating better health.

### Burden of ventricular arrhythmias

The VT incidence was analysed using ICD remote monitoring data, adjusted for observation time, and expressed as events per patient-year. Non-sustained ventricular tachycardia was defined as ≥3 consecutive ventricular beats at a rate >100 b.p.m., lasting <30 s. Sustained VT was defined as VT lasting >30 s or requiring antitachycardia pacing (ATP) therapy or an ICD shock.

The study period was divided into three phases: (i) pre-intervention period: from the day of inclusion to the start of the HIIT programme/control; (ii) intervention period: from the start of the HIIT programme/control to the day of the follow-up CPET; and (iii) post-intervention period, from the day after the follow-up CPET and onwards for 2 months.

To standardize VT detection, the monitoring zone was set at 90% of HR_max_ but not below 133 b.p.m. The rationale for this threshold was to reduce clinically irrelevant alerts caused by sinus tachycardia and to limit the volume of remote monitoring reports. Consequently, VTs with rates between 100 and 132 b.p.m. were not detected. The preset ICD therapy programming remained unchanged unless HR_max_ exceeded the lower limit of the therapy zone.

### Burden of premature ventricular contractions

Ambulatory 48 h Holter monitoring (Philips DigiTrak XT; Philips Medical Systems, Andover, USA) was conducted at baseline and follow-up prior to the CPET, using standard five-electrode placements with three channels of ECG recordings. Automatic analysis (Holter 2010 Plus with Zymed Algorithm; Philips Medical Systems, Andover, USA) followed the manufacturer's default ventricular rules and was supplemented by a manual review, which involved editing classes, evaluating ventricular and atrial events, and scanning for unrecognized ventricular events. Periods with artefacts exceeding 5 min were excluded. Premature ventricular contractions and VT, defined as ≥3 consecutive PVCs, per 24 h was then calculated.

### Echocardiography

A standard transthoracic echocardiographic examination to estimate LV-EF was performed at baseline by a single echocardiographer, using a Vivid E95 scanner (GE Vingmed Ultrasound, Horten, Norway) with a GE M5Sc or 4Vc probe. Another experienced echocardiographer, blinded for intervention identity, performed the analysis.

### Biomarkers

Blood samples were collected in resting conditions and analysed using standard procedures at the Department of Laboratory Medicine at St. Olavs Hospital.

### Statistical methods

Data were analysed using IBM SPPS v29.0.1 (IBM, Armonk, USA) following an intention-to-treat approach. A sample size of 60 patients was determined based on power calculations, providing 80% power to detect a 5 mL/kg/min difference in VO_2_peak at a significance level of *P* < 0.05, using measures of variability from prior data with a similar exercise protocol.^23^ The use of paired data was expected to further enhance statistical power. Similarly, the study had 80% power to detect a 12-percentage-point difference in QoL scores. Because nine SF-36 subscales were analysed, adjustment for multiple comparisons was performed using the Bonferroni method (adjusted *α*=0.006). Given the lack of comparable studies providing variability estimates for arrhythmias, VA burden was designated as secondary endpoint. We anticipated 80% power to detect a 9-percentage-point difference with a significance level of *P* < 0.05 in unpaired analyses, extrapolating from previous research on atrial arrhythmias.^23^

Descriptive statistics are presented as mean ± SD unless otherwise stated. Changes from baseline to follow-up within each group were analysed using the paired-samples *t*-test for normally distributed data and the Wilcoxon signed-rank test for non-normally distributed data. Changes from baseline to follow-up between the groups were analysed using the independent-samples *t*-test or the Mann–Whitney U test, depending on the normality of data distribution. To assess the burden of VA, the mean was chosen as the measure of central tendency despite the data being highly skewed. While the median/interquartile range are typically more robust to skewness, their use here would pose interpretative challenges, as the data distribution would consist entirely of zero values.

All statistical tests were two-tailed and *P* < 0.05 was considered statistically significant.

## Results

**Figure 1:**
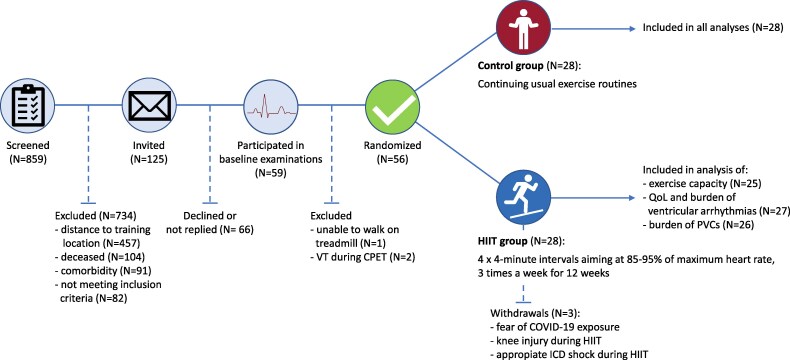
Flowchart of patient inclusion. COVID-19, coronavirus disease 2019; CPET, cardiopulmonary exercise test; HIIT, high-intensity interval training; ICD, implantable cardioverter defibrillator; NSVT, non-sustained ventricular tachycardia; PVC, premature ventricular contraction; QoL, quality of life; VT: ventricular tachycardia.

Of the 859 individuals assessed (81% men), 755 were alive at the time of screening. The reasons for exclusion were residence more than 1 h's drive from the training location (*n* = 457), predefined comorbidities (*n* = 91), and failure to meet the inclusion criteria (*n* = 82). Of the 125 patients invited, 59 accepted and underwent baseline examinations (*Figure 1*). Three patients were excluded after baseline evaluation but before randomization due to VT during CPET (*n* = 2) and inability to perform treadmill exercise (*n* = 1), resulting in 56 patients (82% men, mean age 68.2 ± 8.5 years) being randomized to either the HIIT group or control group (*Table 1*). Both groups were well-matched at baseline for demographics, LV-EF, and New York Heart Association (NYHA) class, device indication, and cardiac arrest history. However, atrial fibrillation/flutter, previous stroke/transient ischaemic attack, and diabetes were more prevalent in the HIIT group. Dual-chamber ICDs were more frequent in the HIIT group, while CRT-Ds were slightly more common in the control group. The use of beta-blockers was similar between groups, while amiodarone use was somewhat higher in the HIIT group. Between baseline and follow-up, five HIIT group patients and six control group patients increased the dosage of one HF drug.

**Table 1:** Patient characteristics and medication use at baseline

	HIIT group (*n* = 28)	Control group (*n* = 28)
Sex		
Male	24 (86)	22 (79)
Female	4 (14)	6 (21)
Age	68 ± 10	68 ± 7
BMI (kg/m^2^)	29.1 ± 4.5	29.0 ± 4.8
Comorbidity		
CAD	18 (64)	20 (71)
Hypertension	12 (43)	11 (39)
Diabetes	9 (32)	4 (14)
Atrial fibrillation/atrial flutter	15 (54)	3 (11)
Persistent	3 (11)	0 (0)
Stroke/TIA	5 (18)	1 (4)
COPD	3 (11)	3 (11)
Revascularization		
Previous CABG	5 (18)	7 (25)
Previous PCI	14 (50)	14 (50)
Previous valve replacement	0 (0)	1 (4)
Left ventricular ejection fraction (%)	36.1 ± 12.3	39.1 ± 11.5
NYHA class		
I	18 (64)	21 (75)
II	8 (29)	6 (21)
III	2 (7)	1 (4)
Aetiology ICD		
CAD	18 (64)	19 (68)
Non-ischaemic DCM	10 (36)	9 (32)
Device type		
Single-chamber ICD	2 (7)	4 (14)
Dual-chamber ICD	14 (50)	10 (36)
CRT-D	12 (43)	14 (50)
ICD/CRT-D implantation indication		
Primary	10 (36)	13 (46)
Secondary	18 (64)	15 (54)
Cardiac arrest survivor	11 (39)	11 (39)
Medication		
Platelet inhibitor(s)	18 (64)	21 (75)
Anticoagulation	16 (57)	5 (18)
Amiodarone	4 (14)	1 (4)
Beta-blocker	26 (93)	26 (93)
ACE-i/ARB/ARNI	25 (89)	25 (89)
MRA	4 (14)	4 (14)
SGLT2 inhibitor	1 (4)	2 (7)
Diuretics	5 (18)	7 (25)
Lipid-lowering agent(s)	23 (82)	23 (82)

Values are *n* (%) or mean ± standard deviation.

ACE-i, angiotensin-converting enzyme inhibitor; ARB, angiotensin receptor blocker; ARNI, angiotensin receptor/neprilysin inhibitor; BMI, body mass index; CABG, coronary artery bypass graft; CAD, coronary artery disease; COPD, chronic obstructive pulmonary disease; CRT-D, cardiac resynchronization therapy-defibrillator; DCM, dilated cardiomyopathy; HIIT, high-intensity interval training; ICD, implantable cardioverter defibrillator; MRA, mineralocorticoid receptor antagonist; NYHA, New York Heart Association; PCI, percutaneous coronary intervention; SGLT-2, sodium–glucose cotransporter 2; TIA, transient ischaemic attack.

In the HIIT group, three patients aborted the training programme. One patient withdrew from the study due to personal concerns of COVID-19 exposure and was consequently excluded from all analyses. A second patient withdrew after sustaining a knee injury during training, preventing further participation. The third patient experienced an appropriate ICD shock during training, leading to exclusion after clinical evaluation by the treating cardiologists. Following the intention-to-treat approach, the latter two patients were included in the analysis of QoL and burden of PVCs and VA but were excluded from the VO_2_peak analysis as they did not perform follow-up CPET.

In total, 53 patients completed all follow-up examinations: 25 in the HIIT group and 28 in the control group. The median monitoring durations were 56 days in the pre-intervention period, 101 days in the intervention period, and 61 days in the post-intervention period.

In the pre-intervention period, one patient randomized to HIIT underwent VT ablation for recurring, drug-refractory symptomatic slow VT. In the control group, one patient underwent VT ablation during the intervention period due to recurrent VA accompanied by ATP therapy and an ICD shock.

### High-intensity interval training participation and related adverse events

In the HIIT group, the average attendance rate for training sessions was 91% (range 73–100%). On average, 91% (range 50–100%) of intervals reached the targeted intensity level. Two patients experienced sustained VT during a training session. Both events were assessed by the treating cardiologists and the internal safety consultant. The first patient experienced a VT that accelerated to ventricular fibrillation, resulting in loss of consciousness and requiring successful termination by an ICD shock. Consequently, this led to exclusion from further HIIT participation. The second patient had an asymptomatic VT episode, successfully managed with ATP. Following a metoprolol dose adjustment from 50 to 100 mg daily, this patient completed the HIIT programme. No further exercise-related arrhythmias were noted in the HIIT group.

Because of the COVID-19 lockdown enforced in Norway during the first half of 2020, four HIIT participants temporarily halted their supervised exercise for 2 months before resuming and completing the programme.

### Primary endpoints

#### Exercise capacity

High-intensity interval training increased VO_2_peak by 1.6 ± 2.1 mL/kg/min (7.0%), from 24.4 mL/kg/min [95% confidence interval (CI), 21.7–27.1] to 26.1 mL/kg/min (95% CI, 23.3–28.9). The control group showed no change in VO_2_peak (0.0 ± 1.3 mL/kg/min), from 24.4 mL/kg/min (95% CI, 21.8–26.9) at baseline to 24.3 mL/kg/min (95% CI, 21.7–26.9) at follow-up. The between-group difference was 1.7 mL/kg/min (95% CI, 0.7–2.6; *P* < 0.001) (*Table 2*). Sensitivity analysis excluding participants with missing data yielded results consistent with the main analysis. Subgroup analysis of the HIIT group showed a 7.9% (1.9 ± 2.1 mL/kg/min) increase in VO_2_peak for patients with an ICD (*n* = 15), and a 5.3% (1.2 ± 2.0 mL/kg/min) rise for those with CRT-D (*n* = 10) (*P* = 0.338 between subgroups).

**Table 2:** Cardiopulmonary exercise test data in the high-intensity interval training and control groups at baseline and at follow-up

	HIIT group (*n* = 25)		Control group (*n* = 28)	
	Baseline	Follow-up	Baseline	Follow-up
Peak oxygen uptake (mL/kg/min)	24.4 ± 6.5	26.1 ± 6.8^a***,^ ^b***^	24.4 ± 6.6	24.3 ± 6.7
Peak oxygen uptake (L/min)	2.21 ± 0.73	2.34 ± 0.73^a***,^ ^b**^	2.12 ± 0.65	2.11 ± 0.65
Oxygen pulse (mL)	16.4 ± 4.0	17.2 ± 4.1^a**^	15.2 ± 3.4	15.5 ± 3.6
Work rate (W)	195 ± 64	197 ± 53	202 ± 66	201 ± 103
V'E/V'O_2_	36.1 ± 7.1	35.4 ± 6.4	33.9 ± 5.8	33.7 ± 5.6
V'E/V'CO_2_	33.3 ± 5.4	32.7 ± 5.5	31.7 ± 4.1	31.9 ± 4.6
Respiratory exchange ratio	1.08 ± 0.06	1.08 ± 0.06	1.07 ± 0.09	1.05 ± 0.06
V'E (L/min)	83.9 ± 25.1	87.0 ± 24.0	76.4 ± 21.0	75.9 ± 21.7
Ventilatory threshold (L)	2.13 ± 0.54	2.09 ± 0.52	2.05 ± 0.47	2.03 ± 0.43
Breathing frequency (/min)	39.5 ± 6.1	41.7 ± 5.3^a**,^ ^b*^	37.4 ± 7.2	37.6 ± 8.0
Maximum heart rate (/min)	138 ± 24	139 ± 24	141 ± 18	137 ± 18^a**,^ ^b*^
Borg RPE	18.3 ± 1.3	18.6 ± 1.1	17.8 ± 1.3	17.8 ± 1.6

Values are mean ± standard deviation.

CPET, cardiopulmonary exercise test; HIIT, high-intensity interval training; RPE, rate of perceived exertion.

^a^Difference from baseline within the group: *P < 0.05, **P < 0.01, ***P < 0.001.

^b^Difference between groups: *P < 0.05, **P < 0.01, ***P < 0.001.

Twenty patients in the HIIT group who achieved ≥90% of intervals within the targeted exercise intensity range had a 7.3% increase in VO_2_peak (1.8 ± 2.2 mL/kg/min). The five patients who completed <90% of intervals within this range showed a 4.6% increase (1.0 ± 1.6 mL/kg/min) (*P* = 0.408 between subgroups).

Maximal oxygen pulse (V'O_2_ peak/HR_peak_) increased significantly in the HIIT group by 0.8 mL (95% CI, 0.3–1.3; *P* = 0.003) but the between-group difference was not statistically significant (0.5 mL; 95% CI, −0.2 to 1.2; *P* = 0.166).

#### Quality of life

From baseline to follow-up, there was a statistically significant between-group difference favouring HIIT in the SF-36 subscale score for health change [+14.8 ± 22.2 points after HIIT vs. −7.1 ± 23.4 points in controls (95% CI, 9.6–34.3; *P* < 0.001)], which remained significant after Bonferroni correction (*α*=0.006). After adjustment for multiple comparisons, no other SF-36 subscale differences between the groups were statistically significant (*Table 3*).

**Table 3:** Quality of life in the high-intensity interval training and control groups at baseline and at follow-up

	HIIT group (*n* = 27)		Control group (*n* = 28)	
SF-36	Baseline	Follow-up	Baseline	Follow-up
Physical functioning	79.8 ± 17.5	85.2 ± 14.6	82.3 ± 17.7	81.8 ± 17.5
Role limitations due to physical health	75.9 ± 33.6	86.1 ± 30.5	70.5 ± 40.9	75.0 ± 39.7
Role limitations due to emotional problems	83.9 ± 32.5	92.6 ± 23.3	82.1 ± 32.1	86.9 ± 29.2
Energy/fatigue	69.8 ± 16.7	69.8 ± 17.7	70.5 ± 20.0	65.5 ± 17.9
Emotional well-being	87.6 ± 12.3	86.1 ± 13.1	82.3 ± 13.3	81.2 ± 13.4
Social functioning	85.2 ± 18.4	89.8 ± 14.3	86.2 ± 19.6	83.9 ± 20.4
Pain	76.7 ± 26.2	77.3 ± 23.3	75.6 ± 23.3	73.3 ± 20.8
General health	64.8 ± 19.0	71.9 ± 14.5	64.5 ± 18.2	65.0 ± 19.0
Health change	62.0 ± 18.8	76.9 ± 18.2^a^	62.5 ± 25.0	55.4 ± 18.5

Values are mean ± standard deviation.

HIIT, high-intensity interval training; QoL, quality of life; SF-36, RAND 36-item Short-Form patient-reported questionnaire.

^a^Difference between groups: *P* < 0.001 (adjusted *α*=0.006 after Bonferroni correction).

### Secondary endpoints

#### Burden of ventricular arrhythmias assessed via remote monitoring data

The prevalence of NSVT and sustained VT events during the pre-intervention, intervention, and post-intervention periods for the HIIT and control group is presented in *Table 4*.

**Table 4:** Prevalence of ventricular arrhythmia events, assessed via remote monitoring data, in HIIT and control group during pre-intervention, intervention, and post-intervention period

	HIIT group (*n* = 27)		Control group (*n* = 28)	
	Number of patients	Number of events	Number of patients	Number of events
Pre-intervention period				
NSVT	7	19	8	49
Sustained VT (requiring ATP or ICD shock)	3 (2)	7 (2)	0 (0)	0 (0)
Intervention period				
NSVT	9	35	11	94
Sustained VT (requiring ATP or ICD shock)	2 (2)	2 (2)	4 (4)	10 (9)
Post-intervention period				
NSVT	5	11	14	58
Sustained VT (requiring ATP or ICD shock)	2 (2)	2 (1)	1 (1)	1 (0)

Sustained VT is defined as ventricular tachycardia lasting >30 s or VT requiring antitachycardia pacing (ATP) or ICD shock.

Pre-intervention period: from inclusion to start of intervention (median 56 days, range 35–126 days).

Intervention period (median 101 days, range 81–213 days).

Post-intervention period: from follow-up to 2 months after (median 61 days, range 61–61 days).

HIIT, high-intensity interval training; NSVT, non-sustained ventricular tachycardia; VT, ventricular tachycardia.

In both groups, the distribution of events was highly skewed, with a few patients accounting for most events. In the HIIT group, 23 of 27 patients showed no change in sustained VT incidence (0 events per patient-year). Three patients showed a decrease (−15.3, −7.5, and −2.7 events per patient-year, respectively) and one had an increase (+3.6 events per patient-year), resulting in a mean decrease of −0.8 events per patient-year for the entire group. In the control group, 24 of 28 patients had no change (0 events per patient-year), while 4 patients experienced an increase (+2.4, +3.2, +4.2, and +17.7 events per patient-year, respectively), yielding a mean increase of +1.0 events per patient-year for the group (*Table 5*). This between-group difference was statistically significant (*P* = 0.037).

**Table 5:** Change in the incidence of ventricular arrhythmia (events per patient-year), assessed via remote monitoring data, in the high-intensity interval training and control groups from baseline to follow-up and from baseline to 2 months post-follow-up

	Change from baseline to follow-up	Group difference (HIIT vs. control) at follow-up	Change from baseline to 2 months post-follow-up	Group difference (HIIT vs. control) at 2 months post-follow-up
**NSVT**				
** HIIT group (*n* = 27)**	0.6 ± 13.2	−0.5	−1.1 ± 4.1	−3.1
** Control group (*n* = 28)**	1.0 ± 22.3		2.0 ± 17.8	
**Sustained VT**				
** HIIT group (*n* = 27)**	−0.8 ± 3.4	−1.8*	−0.6 ± 2.8	−0.8
** Control group (*n* = 28)**	1.0 ± 3.5		0.2 ± 1.1	

Values are mean ± standard deviation.

Sustained VT is defined as ventricular tachycardia lasting >30 s or VT requiring antitachycardia pacing (ATP) or an ICD shock.

ICD, implantable cardioverter defibrillator; HIIT, high-intensity interval training; NSVT, non-sustained ventricular tachycardia; VT, ventricular tachycardia.

^*^ *P* < 0.05.

In the 2 months following the HIIT programme, there was a trend towards lower event per patient-year rates in the HIIT group, but with no statistically significant differences from baseline between groups for NSVT (*P* = 0.059) or sustained VT (*P* = 0.177).

#### Burden of premature ventricular contractions assessed via Holter monitoring

No significant changes in PVCs or VT incidence were observed from baseline to follow-up in either the HIIT group or control group (see Supplementary material online, *Table S1*). One patient in the HIIT group had missing follow-up Holter monitoring data.

#### Biomarkers

*N*-terminal pro-B-type natriuretic peptide (NT-proBNP) showed a non-significant 2.5% decrease in the HIIT group (881 ± 1190 to 859 ± 1348 ng/L; *P* = 0.052) and a 14% increase in the control group (858 ± 1491 to 974 ± 2282 ng/L; *P* = 0.402); the between-group difference was also non-significant (*P* = 0.341). After Bonferroni correction for eight biomarkers (adjusted *α*=0.006), no biomarkers showed statistically significant changes (see Supplementary material online, *Table S2*).

#### Adverse events unrelated to the exercise programme

During the pre-intervention period, one patient randomized to the HIIT group required replacement of an ICD lead due to lead failure.

During the intervention period, one control group patient developed angina pectoris and underwent coronary angiography for revascularization, while another control group patient was hospitalized for gastrointestinal bleeding.

In the post-intervention period, one HIIT group patient fell on the ice, resulting in a chronic subdural haemorrhage that required surgical evacuation. Another HIIT group patient experienced an ICD lead fracture, necessitating extraction and simultaneously upgrade to a CRT-D. The lead defect was considered unrelated to the trial. Lastly, one HIIT group patient was hospitalized for asymptomatic atrial flutter, treated with cardioversion.

## Discussion

The present study demonstrates that a supervised 12-week HIIT programme improves exercise capacity and QoL in patients with an ICD and CAD or non-ischaemic DCM. Clinically relevant VTs were observed during both exercise testing and intervention. Overall, fewer sustained VT events occurred in the HIIT group.

### Exercise capacity

In this study, 12 weeks of HIIT improved VO_2_peak with 7.0% (1.6 mL/kg/min within the HIIT group; 1.7 mL/kg/min compared with controls). Cardiovascular fitness, directly measured as VO_2_peak, is a robust independent predictor of mortality among patients with ischaemic HF and DCM.^24,25^ A 6% increase in VO_2_peak is associated with a clinically substantial decrease in cardiovascular mortality and hospitalization for HF.^26^ The relative increase in VO_2_peak in our study was slightly lower compared with other studies. Berg *et al*.^27^ showed that a comprehensive cardiac rehabilitation programme combined with psycho-educational follow-up in ICD patients enhanced VO_2_peak with 2.1 mL/kg/min compared with controls. Dougherty *et al*.^28^ evaluated the effects of a moderately strenuous home-based HIIT programme in ICD patients, observing an increase in VO_2_peak by 2.8 mL/kg/min in the exercise group compared with controls. In a pooled meta-analysis of six trials with moderate to high exercise intensity in patients with HF and an ICD, Pandey *et al*.^12^ found that exercise training improved VO_2_peak by a weighted mean difference of 1.98 mL/kg/min in comparison to controls. Relative to the six studies included in that meta-analysis, our study included patients with a higher mean age but a better NYHA functional class. We included patients with both single- and dual-chamber ICDs, as well as those with CRT-D.

In the HIIT group, patients with an ICD experienced a slightly greater increase in VO_2_peak vs. those with a CRT-D (7.9 vs. 5.3%). This difference, although not statistically significant, may be partially explained by some CRT-D patients having an upper tracking rate set too low, leading to the loss of biventricular stimulation at peak exercise, a critical moment when such stimulation would have been most beneficial. The study protocol did not include adjustment of the upper tracking rate for HR_max_. In contrast, Belardinelli *et al*.^29^ found a more notable exercise-induced improvement in cardiovascular fitness and QoL in HF patients with CRT-D compared with those with ICD.

While statistical significance was not reached, we observed that patients completing ≥90% of intervals within the targeted exercise intensity level had a slightly greater VO_2_peak increase vs. those completing <90% of intervals (7.3 vs. 4.6%). Similarly, Isaksen *et al*.^13^ observed a greater numerical increase in VO_2_peak among patients exercising with a mean Borg’s RPE of ≥16, compared with those with an RPE of <16. These results suggest that maintaining a higher exercise intensity further enhances cardiovascular fitness improvements in this patient group as well.

### Quality of life

Patient-reported outcome measurements, such as the SF-36 questionnaire, are acknowledged as important tools for evaluating patient well-being. Implantable cardioverter defibrillator patients have demonstrated lower health-related QoL than the general population.^30^ This can be attributed to experiencing device therapy, fear of shocks, adverse events associated with the ICD and the implantation procedure, as well as restrictions on daily life stemming from having an ICD and the underlying condition. Poor QoL is associated with a greater risk of mortality in patients with an ICD.^31^ Our findings suggest that HIIT may offer a promising approach to improve QoL in this population. Between-group differences were observed across most SF-36 domains. However, after adjustment for multiple comparisons across the nine subscales, only the improvement in health change remained statistically significant. Although the remaining differences did not reach the adjusted threshold for significance, patients performing HIIT demonstrated numerical improvements in all but one of the SF-36 domains compared with controls, indicating a consistent pattern favouring the intervention.

Documentation on improvements in QoL after HIIT in ICD patients is limited. An RCT of comparable size, but with a moderate exercise intensity level at 60% of VO_2_peak, demonstrated marked enhancements in the Minnesota Living with Heart Failure Questionnaire score, especially among the patients with CRT-D.^29^ However, to our knowledge, no RCT involving HIIT with a target HR exceeding 85% of HR_max_ has investigated its impact on QoL in ICD patients. While conventional endpoints in HF studies have traditionally revolved around reduced hospitalization and mortality, it is increasingly emphasized that improvements in QoL, symptoms, and physical function should be considered important endpoints.^32^ In this context, the robust improvement in health change, together with consistent directional improvements across most QoL domains, supports a potential role for HIIT as an adjunct to standard care in selected ICD patients. These findings should, however, be interpreted with caution, given the multiplicity of analyses.

### Arrhythmias

In this study, 27 patients participated in the HIIT programme, totalling 577 h of exercise. The overall incidence of sustained VT was low in both groups, and all ATPs and shocks were considered appropriate. One episode of VT treated with ATP and one ICD shock occurred during HIIT sessions. In addition, 2 of the 59 patients undergoing baseline CPET met the study’s exclusion criteria due to VT during testing.

A prevalent challenge lies in the uncertainty of when exercise increases the risk of malignant arrhythmia or sudden cardiac death (SCD) vs. when it provides long-term benefits in high-risk individuals. Studies on ostensibly healthy individuals imply an increased risk of SCD during or immediately after intense physical activity.^33^ Similarly, patients with an ICD show a heightened risk of VA associated with physical exercise.^34^ These reports are consistent with our observations, suggesting a possible causal link between exertion and arrhythmia onset.

In parallel, previous meta-analyses have reported a favourable impact of exercise on ICD therapy.^12,35^ Comparing the incidence of ICD shocks in our study to other trials is challenging due to differences in sample sizes, follow-up durations, comorbidity levels, HF severity, as well as variations in exercise protocols, including intensity levels and duration. Although we observed a statistically significant between-group difference in sustained VT burden favouring HIIT, most patients in both groups had no sustained VT events at baseline or follow-up, while a few patients accounted for most sustained VT events, suggesting limited clinical relevance for the overall cohort. Additionally, the minor imbalance in amiodarone use may have influenced the arrhythmic outcomes. None of the four HIIT group patients on amiodarone experienced VT events, whereas the single amiodarone user in the control group had one sustained VT episode. Also, one patient in the HIIT group discontinued amiodarone during the intervention. These factors should therefore be considered when interpreting the results.

Despite the heightened short-term risk, epidemiological studies suggest that the overall positive effect of physical exercise clearly outweigh the potential risk, both in assumed healthy individuals and those with cardiovascular disease.^36,37^ This is consistent with the strong association between moderate to high levels of physical exercise and a substantial decrease in all-cause mortality.^38^ However, the low number of arrhythmic events in the present study neither supports nor refutes these associations in this high-risk population.

The most common triggers for inappropriate shocks are atrial fibrillation, supraventricular tachycardia, and abnormal sensing.^39^ Strenuous exercise can induce any of these triggers. While the lack of inappropriate shocks in this and other studies is reassuring, it is important to address this risk before initiating HIIT in ICD patients. The absence of inappropriate shocks in our study may also reflect advances in device technology, including improved algorithms for detecting true VA, and the trend towards more conservative ICD programming.^40^

Given the exclusion criteria, heterogeneity of data and limited statistical power in the present study, no conclusions can be drawn regarding potential antiarrhythmic benefits of HIIT. Nevertheless, the occurrence of VT, with or without ICD therapy, during CPET and HIIT underscores the need for individual patient evaluation before recommending high-intensity exercise to individuals with an ICD.

Larger, randomized trials with longer follow-up are needed to determine the short- and long-term benefits for HIIT. For ICD patients considering participation in HIIT, a maximal exercise test is advisable to assess functional capacity, identify those at higher risk to exercise-induced VA, and determine HR_max_ for potential adjustment of ICD therapy zones.

### Limitations and strengths

The single-centre design, modest sample size, and relatively short intervention period are important limitations. Recruitment ended before reaching the planned target of 60 patients due to insufficient enrolment from the predefined patient population, making the study slightly underpowered for the primary endpoints. Nevertheless, the randomized controlled design strengthens the validity of our findings. The low inclusion rate was primarily attributable to the predefined exclusion criteria, which should be considered when generalizing the results to a broader clinical setting. Among these criteria, we regard the comorbidity restrictions as the most important, particularly the exclusion of patients who experienced VT during baseline CPET. The study also excluded patients with other diagnoses warranting ICD placement, such as channelopathies, ARVC, or myocarditis.

All patients who underwent ICD implantation at our clinic during the specified period were screened for inclusion, ensuring thorough patient selection. Of 859 patients screened, 734 were excluded, which may introduce selection bias and limit external validity. However, long travel distance to the training facility, accounted for most exclusions, affecting 62% (*n* = 457) of patients who were alive and otherwise eligible. This factor is unlikely to substantially affect the generalizability of the findings. Offering supervised HIIT at local hospitals or community-based training facilities, rather than exclusively at our implantation centre, would likely have increased enrolment.

Given that nearly half of the invited patients either declined participation or did not respond, volunteer bias cannot be ruled out, as patients more motivated and confident in their ability to exercise may have been more inclined to participate.

Despite these exclusion criteria, 84% of patients (*n* = 47) in the study had ≥2 comorbid conditions, and 52% (*n* = 29) had ≥3 comorbid conditions, making the study cohort similar to the general ICD population.^41,42^ Although women were underrepresented in this study, they comprised 18% of study participants, which closely mirrors the 19% of women among all patients assessed for eligibility. The sex and age profile also corresponded well with previous studies.^43,44^ Together, these aspects collectively influence the broader applicability of our results. However, as in many small, randomized trials, the limited sample size likely contributed to certain baseline imbalances, including differences in atrial fibrillation and to a lesser degree CRT-D use, which may have influenced clinical and arrhythmic outcomes and should be considered when interpreting the results.

A common challenge in exercise-related research is the lack of blinding for patients concerning the exercise intervention, potentially impacting the self-reported QoL evaluation. Additionally, participation in group-based exercise led by an exercise physiologist in a setting with other individuals sharing similar health conditions may have introduced a social desirability bias, which in turn could have positively influenced QoL outcomes and adherence to the exercise programme. However, this setting could better resemble real-life scenarios, potentially enhancing the applicability of the findings.

Another limitation was the absence of active monitoring in the control group, as their physical activity was self-reported at baseline and follow-up, and they were only instructed to maintain their habitual activity levels if desired and avoid new structured exercise. Despite these instructions, at least two control group participants reported non-adherence and independently undertook an HIIT programme similar to the intervention. This contamination may have attenuated the observed effect of HIIT. Additionally, three patients withdrew from the HIIT programme, though two were followed for all endpoints except VO_2_peak, and the final analysis was conducted using the intention-to-treat approach, maintaining the integrity of the results.

As the study was not powered to assess safety outcomes, caution is warranted when applying these findings to similar patient groups.

Finally, a major strength of the study was the high adherence to the HIIT protocol, ensuring an impactful intervention. Both the CPET and the HIIT sessions were conducted exclusively on a treadmill, which is relevant because untrained patients typically achieve 5–20% lower VO_2_peak on bicycle ergometers compared with treadmills.^45^

## Conclusions

A 12-week supervised HIIT programme at 85–95% of maximum heart rate significantly improved exercise capacity and perceived health change in patients with an ICD due to CAD or non-ischaemic DCM. Although improvements in other SF-36 QoL domains did not reach statistical significance after adjustment for multiple comparisons, the consistent numerical trends suggest a favourable impact of HIIT on overall well-being.

The low number and uneven distribution of arrhythmic events limit firm conclusions regarding the effect of HIIT on VA. Nonetheless, we observed clinically relevant VA during exercise, confirming that strenuous activity such as HIIT may carry an arrhythmic risk in this high-risk population. Still, integrating HIIT into the routine of selected ICD patients can enhance cardiovascular fitness and overall well-being, aligning with prior research in other cardiac conditions.^46^ However, the risk of exercise-induced VA should be evaluated individually.

The present study demonstrates the feasibility of conducting larger, long-term trials to determine the optimal intensity and duration of exercise training for patients at high risk for VA.

## Supplementary Material

oeag058_Supplementary_Data

## Data Availability

The data underlying this article may be shared upon reasonable request to the corresponding author, subject to potential limitations related to patient consent and data anonymization.
