# Supplementary material for: Effect of high-intensity interval training on peak oxygen uptake, quality of life, and ventricular arrhythmias in patients with an implantable cardioverter defibrillator: a randomized controlled trial
Source: Eur Heart J Open. 2026 Apr 13;6(2):oeag058. doi: 10.1093/ehjopen/oeag058 (PMC13089605; doi:10.1093/ehjopen/oeag058)
Supplement: oeag058_Supplementary_Data [file oeag058_supplementary_data.zip › Table S1 PVC burden.docx]

|  | | | | | | | |
| --- | --- | --- | --- | --- | --- | --- | --- |
|  | **HIIT group (n=26)** | |  | | **Control group (n=28)** | |  |
|  | **Baseline** | **Follow-up** | |  | **Baseline** | **Follow-up** |  |
| PVC | 405 (0-2272) | 245 (1-2496) | |  | 1023 (4-20538) | 558 (4-14075) |  |
| VT | 0.3 (0-22) | 0 (0-6) | |  | 0.6 (0-42) | 1.1 (0-47) |  |
| **Table S1. Incidence of PVC and VT (per 24 hours), assessed via Holter monitoring,**  **in HIIT and control group at baseline and follow-up**  Values are median (minimum-maximum value).  VT is defined as ≥3 consecutive PVCs.  HIIT, high-intensity interval training; PVC, premature ventricular contraction; VT, ventricular tachycardia. | | | | | | | |
