# Supplementary material for: Effect of high-intensity interval training on peak oxygen uptake, quality of life, and ventricular arrhythmias in patients with an implantable cardioverter defibrillator: a randomized controlled trial
Source: Eur Heart J Open. 2026 Apr 13;6(2):oeag058. doi: 10.1093/ehjopen/oeag058 (PMC13089605; doi:10.1093/ehjopen/oeag058)
Supplement: oeag058_Supplementary_Data [file oeag058_supplementary_data.zip › Table S2 Blood analysis.docx]

|  | | | | | | |  |
| --- | --- | --- | --- | --- | --- | --- | --- |
|  | **HIIT group (n=27)** | |  | | **Control group (n=28)** | | |
|  | **Baseline** | **Follow-up** | |  | **Baseline** | **Follow-up** | |
| NT-proBNP (ng/L) | 881 ± 1190 | 859 ± 1348 | |  | 858 ± 1491 | 974 ± 2282 | |
| Troponine T (ng/L) | 18 ± 10 | 19 ± 12 | |  | 15 ± 8 | 16 ± 7 | |
| Total cholesterol (mmol/L) | 4.0 ± 0.9 | 3.9 ± 0.7 | |  | 4.6 ± 1.4 | 4.5 ± 1.6 | |
| HDL (mmol/L) | 1.3 ± 0.4 | 1.2 ± 0.3 | |  | 1.4 ± 0.4 | 1.3 ± 0.4 | |
| LDL (mmol/L) | 2.3 ± 0.7 | 2.2 ± 0.7 | |  | 2.7 ± 1.3 | 2.8 ± 1.4 | |
| Triglycerides (mmol/L) | 1.63 ± 0.94 | 1.43 ± 0.78 | |  | 1.58 ± 0.74 | 1.43 ± 0.78 | |
| Creatinine (mmol/L) | 85 ± 14 | 86 ± 12 | |  | 85 ± 22 | 86 ± 20 | |
| eGFR (mL/min/1.73 m^2^) | 77 ± 13 | 76 ± 12 | |  | 74 ± 17 | 74 ± 17 | |
| **Table S2. Biomarkers in HIIT and control group at baseline and at follow-up.**  Values are mean ± standard deviation. | | | | | |  |  |

eGFR, estimated glomerular filtration rate; HDL, high-density lipoprotein; HIIT, high-intensity interval training; LDL, low-density lipoprotein; NT-proBNP, N-terminal pro-B-type natriuretic peptide.
